# Origin and Evolution of Dendritic Epidermal T Cells

**DOI:** 10.3389/fimmu.2018.01059

**Published:** 2018-05-14

**Authors:** Yoichi Sutoh, Rania Hassan Mohamed, Masanori Kasahara

**Affiliations:** ^1^Division of Biobank and Data Management, Iwate Tohoku Medical Megabank Organization, Iwate Medical University, Shiwa-gun, Japan; ^2^Department of Biochemistry, Faculty of Science, Ain Shams University, Cairo, Egypt; ^3^Department of Pathology, Faculty of Medicine, Hokkaido University, Sapporo, Japan

**Keywords:** dendritic epidermal T cell, γδ T cell, *Skint1*, skin, epidermis, intraepithelial lymphocyte

## Abstract

Dendritic epidermal T cells (DETCs) expressing invariant Vγ5Vδ1 T-cell receptors (TCRs) play a crucial role in maintaining skin homeostasis in mice. When activated, they secrete cytokines, which recruit various immune cells to sites of infection and promote wound healing. Recently, a member of the butyrophilin family, *Skint1*, expressed specifically in the skin and thymus was identified as a gene required for DETC development in mice. *Skint1* is a gene that arose by rodent-specific gene duplication. Consequently, a gene orthologs to mouse *Skint1* exists only in rodents, indicating that *Skint1*-dependent DETCs are unique to rodents. However, dendritic-shaped epidermal γδ T cells with limited antigen receptor diversity appear to occur in other mammals. Even lampreys, a member of the most primitive class of vertebrates that even lacks TCRs, have γδ T-like lymphocytes that resemble DETCs. This indicates that species as divergent as mice and lampreys share the needs to have innate-like T cells at their body surface, and that the origin of DETC-like cells is as ancient as that of lymphocytes.

## Introduction

In the mouse epidermis, ~95% of cells are keratinocytes, and the remaining 5% are immune cells such as Langerhans cells and T cells. The majority of epidermis-resident T cells are γδ T cells with a highly dendritic shape, extending dendrites both basally and apically; along with Langerhans cells, these γδ T cells form an interdigitating network within the layers of the epidermis (1). Therefore, they are called dendritic epidermal T cells (DETCs) (2). Interestingly, ~90% of DETCs express an invariant Vγ5Vδ1 T-cell receptor (TCR) without any junctional diversity [also called Vγ3Vδ1 TCR according to the nomenclature of Garman et al. (3)]. It is thought that DETCs recognize a limited set of “stress antigens” induced on damaged or dysregulated keratinocytes through their invariant TCRs in a major histocompatibility complex (MHC)-independent manner (4). Although the molecular identity of “stress antigens” recognized by Vγ5Vδ1 TCR remains unknown, co-stimulatory molecules on DETCs, which synergistically amplify TCR signals, have been identified. The most important among them are the junctional adhesion molecule-like protein JAML (5), CD100 (also known as semaphorin 4D) (6), and NKG2D receptors (7, 8), which interact with the coxsackie and adenovirus receptor, plexin B2, and a group of stress-inducible MHC class I-like molecules known as NKG2D ligands (9, 10), respectively.

Once activated, DETCs retract their dendrites, adopt a rounded shape, and secrete a range of cytokines, chemokines, and tissue-specific growth factors, leading to increased keratinocyte proliferation and recruitment of infiltrating leukocytes, thereby promoting wound healing and immune surveillance in the skin. Among the cytokines secreted by DETCs is insulin-like growth factor 1, which aids wound healing by preventing apoptosis of keratinocytes and DETCs themselves in an autocrine manner (11). Although controversial, one recent study has shown that a subset of DETCs secretes interleukin-17A that induces production of antimicrobial peptides such as β-defensin 3 and regenerating islet-derived protein 3γ (12). The latter peptide also induces keratinocyte proliferation and differentiation after skin injury (13), indicating that interleukin-17 plays a role in both infection control and epithelial proliferation at wound sites.

In the thymus of fetal mice, γδ T cells with particular γ and δ chains appear sequentially in discrete waves. DETC progenitors bearing Vγ5Vδ1 TCRs appear at embryonal days 14–16 (14, 15), after which they home to the epidermis. On the other hand, terminal deoxynucleotidyl transferase (TdT), which generates junctional diversity in V(D)J recombination by attaching additional nucleotides (so-called N nucleotides) at the 3′-end of gene segments in a template-independent way (15), is not expressed in fetal thymus and starts to be expressed 4 days after birth (14). Forced expression of TdT in fetal thymus produces DETCs expressing Vγ5Vδ1 TCRs with junctional diversity, which populate the epidermis of newborn mice (16). These DETCs, however, gradually disappear after birth, unlike normal DETC. Therefore, it appears that TCR specificity is not required for epidermal migration of DETC progenitors, but important for renewing and sustaining DETCs in the epidermis. Likewise, epidermis-resident T cells in TCRδ-deficient mice, which mainly express variable αβ TCRs, are gradually lost and not retained over a lifetime (17), again indicating the importance of TCR specificity for the maintenance of epidermal T cells.

Dendritic epidermal T cells have been identified in rodents such as mice (18) and rats (19, 20). However, little is known about the origin and evolution of DETCs. Recently, a gene essential for DETC development, named *Skint1* (selection and upkeep of intraepithelial T cells protein 1), was identified in mice (21, 22). Interestingly, the *Skint1*-like (*SKINT1L*) gene is inactivated in humans, raising the possibility that this inactivation might be responsible for the deficiency of DETCs in humans. In this review, we summarize the evolution of the *SKINT* gene family and its implications for the origin and evolution of DETCs. Available evidence indicates that *Skint1*-dependent DETCs are unique to rodents. However, if we define DETCs more broadly as dendritic-shaped epidermal γδ T cells with limited antigen receptor diversity, they seem to occur in other mammals. Indeed, even lampreys, a member of the most primitive class of vertebrates equipped with lymphocytes, have DETC-like cells, suggesting that DETCs also exist in jawed vertebrates other than mammals.

## *SKINT1* and the *SKINT* Gene Family

The epidermis of FVB/N mice from Taconic Farms (FVB/N Tac) lacks Vγ5Vδ1 DETCs, while γδ T-cell repertoires in other tissues are normal (23). *Skint1* was identified as a gene responsible for this depletion of canonical DETCs (21). SKINT1 is a membrane-bound immunoglobulin (Ig) superfamily protein made up of an Ig variable (IgV) domain, an Ig constant domain and three transmembrane regions. It is specifically expressed by thymic epithelial cells and skin keratinocytes. The *Skint1* gene of FVB/N Tac mice contains a premature termination codon in the region coding for the segment between the second and third transmembrane regions. In *Skint1*-deficient mice, Vγ5Vδ1 T cells are present in fetal thymus in comparable numbers to wild-type FVB/N mice at embryonic days 14–16.5, but the production of mature Vγ5Vδ1 T cells migrating to the epidermis is impaired because of defective thymic selection of Vγ5Vδ1 T cells (23). The complementarity determining region 3-like loop in the IgV domain of SKINT1 molecules appears important for this selection (24). However, it is not known whether SKINT1 or the SKINT1 molecular complex interacts with Vγ5Vδ1 TCR itself or an as yet uncharacterized molecule expressed uniquely on DETC progenitors (22).

*Skint1* is a member of the *Skint* gene family. Mice have 11 *Skint* genes (paralogs) designated *Skint1* to *Skint11*, coding for structurally related proteins with similar, though not identical, expression patterns (21). These paralogs appear to have distinct functions. Thus, neither *Skint2* nor *Skint7* can compensate for the loss of *Skint1* function in reaggregate fetal thymic organ culture (22). Furthermore, mice selectively deficient in epidermal *Skint1* expression show only a minor delay in wound healing compared to mice deficient in *Skint3* or *Skint9*, suggesting that *Skint1* is mainly involved in the maturation of DETC progenitors in the thymus, and that *Skint3* and *Skint9* play a more important role in mediating DETC activation in the epidermis (25). These observations suggest that *Skint* paralogs have undergone functional specialization, with only *Skint1*serving as a selecting component for Vγ5Vδ1 T cells.

## Evolution of the *SKINT* Gene Family

**Figure 1:**
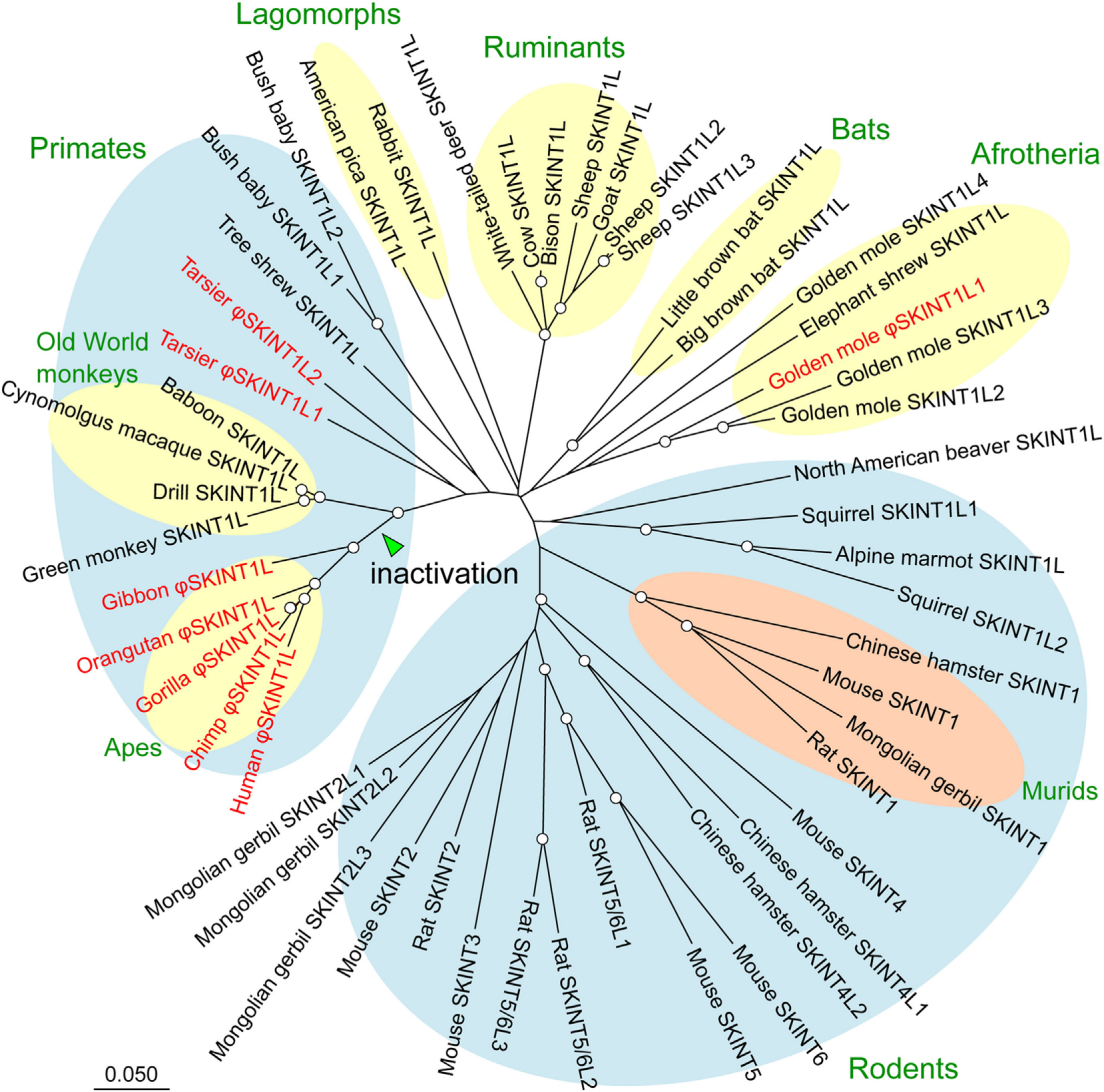
Phylogenetic tree of the SKINT1 subfamily. SKINT1 subfamily sequences were retrieved from the NCBI database (WGS and nr databases) using the mouse Skint1 sequence as a query. Deduced amino acid sequences of SKINT1 subfamily proteins were aligned, and the phylogenetic tree was constructed using the MUSCLE and neighbor-joining programs implemented in MEGA7, respectively. The distance matrix was obtained by calculating p-distances for all pairs of sequences. Gaps were excluded using the pairwise-deletion option. The reliability of branching patterns was assessed by bootstrap analysis (1,000 replications). Pseudogenes are indicated by φ and shown in red. Nodes supported by bootstrap values over 80% are indicated by open circles. *Skint7* to *Skint11* genes of mice are not shown as they are the members of the *SKINT7* or *SKINT9* subfamily.

The *SKINT* gene family, which occurs only in placental mammals, is a member of a larger gene family known as the butyrophilin family (26–28). It comprises three subfamilies, *SKINT1, SKINT7*, and *SKINT9* (29). Figure 1 shows the phylogenetic tree of *SKINT1* subfamily genes in placental mammals. Whereas mice and rats have multiple copies of these subfamily genes, most mammals have either a single copy of *SKINT1* genes known as *SKINT1L* or altogether lack this subfamily. The branching pattern of the phylogenetic tree indicates that mouse *Skint1* to *Skint6* genes emerged by rodent-specific gene duplication from an *SKINT1L* gene. Therefore, non-rodents do not have an *SKINT* gene orthologs to mouse *Skint1*. Actually, a gene ortholog to mouse *Skint1* exists only in some rodents, specifically family Muridae or murids such as mice, rats, hamsters, and Mongolian gerbils. Coupled with the finding that *Skint* paralogs in mice have undergone functional specialization and have distinct functions (22, 25), these observations indicate that authentic *Skint1* genes are unique to rodents, more precisely murids.

A notable feature of *SKINT1L* genes is that they are absent in a number of species such as elephants, sloths, armadillos, alpacas, horses, cats, dogs, and ferrets. They are also inactivated in several mammalian species. Thus, all the hominoids including humans, great apes (chimpanzees, gorillas, and orangutans), and lesser apes (gibbons) have *SKINT1L* genes inactivated by multiple mutations (29). One of the mutations, the stop codon located at the ninth residue of the IgV domain, is shared by all the hominoid sequences. Because Old World monkeys such as olive baboons, green monkeys, cynomolgus macaques, and rhesus macaques do not have this mutation, and their *SKINT1L* genes are apparently functional, this stop codon was most likely responsible for the initial inactivation of the hominoid *SKINT1L* gene (29). Tarsiers, pigs, and whales also have inactivated *SKINT1L* genes. Therefore, *SKINT1L* appears to have been lost or inactivated independently in multiple mammalian lineages.

## DETCs in Other Mammals

The observation that the *Skint1* gene essential for DETC development exists only in rodents (Figure 1) indicates that *Skint1*-dependent DETCs are unique to rodents. Indeed, rats are the only species in which the presence of cells quite similar to mouse DETCs has been unambiguously demonstrated (20). In the rat epidermis, αβ T cells occupy only 0.03–0.24% of CD3^+^ cells, indicating that the vast majority of T cells are γδ T cells (30). Immunostaining with a specific antibody against γδ TCR revealed that the rat epidermis abundantly contains γδ T cells with dendritic morphology (19). Vγ and Vδ chains expressed on these γδ T cells are very similar to mouse Vγ5 and Vδ1, with 92 and 95% amino acids sequence identity, respectively, and lack junctional diversity.

In cattle, a representative member of γδ-high species, more than 80% of skin T cells, of which at least 44% are γδ T cells, occur in the superficial 0.5 mm of the dermis, with only 3% in the epidermis (31). Thus, distribution of skin T cells differs from that in mice. Skin-resident bovine T cells are irregular in shape and frequently have a flattened outline with wavy cytoplasmic projections. Furthermore, although the information on TCR γ- and δ-chain usage in epidermal γδ T cells is not available, skin-resident γδ T cells as a whole predominantly use Vγ3 and Vγ7 while the Vδ usage is diverse (32). Therefore, it is possible that cattle have DETCs broadly defined as dendritic-shaped epidermal γδ T cells with limited antigen receptor diversity.

In humans, Vδ1 T cells, which express different Vγ elements, are the major γδ T cell subset preferentially homing to epithelial tissues such as skin and intestine (33, 34). In the skin, Vδ1 T cells reside mainly in the dermis but are also found in the epidermis. Like mouse DETCs, activated Vδ1 T cells produce insulin-like growth factor 1 and promote wound healing (33). They also exert cytotoxic responses against tumors. Therefore, they seem to perform functions similar to those of rodent DETCs. However, because human epidermal Vδ1 T cells do not have a distinctive dendritic shape, it seems inappropriate to call them DETCs.

In non-human primates, the possibility of the existence of DETCs was examined in cynomolgus macaques (crab-eating macaques) because, unlike humans which have inactivated *SKINT1L*, macaques have a single copy of structurally intact *SKINT1L* (29). Like its mouse counterpart, macaque *SKINT1L* is expressed in the thymus and skin, and the basal and suprabasal layers of the macaque epidermis contain a population of dendritic-shaped γδ T cells. Macaque epidermal T cells predominantly expressed Vγ10Vδ1 TCRs, but both Vγ and Vδ chains displayed junctional diversity. Also, expression of macaque Vγ10 was not restricted to epidermal lymphocytes. Therefore, it was concluded that macaques do not have rodent-type DETCs (29), but it is possible that they have DETCs defined as dendritic-shaped epidermal γδ T cells with limited antigen receptor diversity.

In summary, DETCs that are selected by SKINT1 molecules and display an invariant γδ TCR are unique to rodents, but DETCs in a broad sense appear to occur in other mammals, although more detailed investigation is required to draw definitive conclusions.

## Origin and Evolution of DETCs

Recent work has uncovered that the epidermis of lampreys, a member of jawless vertebrates, contains dendritic-shaped γδ T-like cells with limited antigen receptor diversity reminiscent of DETCs (35).

Jawless vertebrates represented by lampreys and hagfish are the most primitive class of vertebrates equipped with adaptive immunity; accumulated evidence indicates that lymphocytes forming the cornerstone of adaptive immunity emerged in a common ancestor of jawed and jawless vertebrates. Interestingly, instead of TCRs and B-cell receptors (BCRs), jawless vertebrates use members of the leucine-rich repeat (LRR) family of proteins known as variable lymphocyte receptors (VLRs) for antigen recognition (36–39). Like gnathostome antigen receptors, VLRs are clonally expressed on lymphocytes. The diversity of VLRs, which is assumed to be comparable to that of TCRs and BCRs, is generated during lymphocyte development by assembly of multiple LRR modules with highly variable sequences through a gene-conversion-like mechanism. Jawless vertebrates have three major populations of lymphocytes distinguished by expression of distinct types of VLRs known as VLRA, VLRB, and VLRC. VLRB^+^ cells resemble gnathostome B cells; when activated by specific antigen, they undergo clonal proliferation and secrete VLRB molecules as antibodies. On the other hand, VLRA^+^ and VLRC^+^ cells develop in lympho-epithelial thymus-like structures named thymoids, do not secrete VLR molecules, and resemble gnathostome T cells in gene expression profiles and responses to mitogens. Of the two T-like lymphocyte populations, VLRC^+^ cells resemble γδ T cells in gene expression profiles in that they express the SRY-box containing gene 13 encoding a fate-determining transcription factor important for γδ T-cell lineage determination and interleukin-17. They also resemble gnathostome γδ T cells in tissue localization; VLRC^+^ cells in lampreys are distributed predominantly in the epithelium of skin, intestine, and gill (35). In the epidermis, VLRC^+^ cells are ~8 times more abundant than VLRA^+^ cells and display dendritic morphology. Furthermore, the diversity of VLRC receptors in epidermal lymphocytes is markedly reduced compared to that in kidneys and blood.

The existence of DETC-like lymphocytes in the epidermis of lampreys indicates that the strategy of deploying γδ-like T cells to epithelia was adopted in a vertebrate ancestor and has been maintained in many vertebrate animals.

## Concluding Remarks

*Skint1*-dependent DETCs appear unique to rodents such as mice and rats. However, DETCs broadly defined as dendritic-shaped epidermal γδ T cells with limited antigen receptor diversity appear to exist in other mammals. The presence of DETC-like cells in lampreys suggests that DETCs also occur in jawed vertebrates other than mammals. In animals in which *Skint1* is absent, other members of the butyrophilin family may perform equivalent functions. Also, “stress antigens” recognized by DETCs most likely differ from species to species. This difference, along with the difference in the butyrophilin members used for selection, may account for the observation that γδ TCRs on DETCs are invariant in rodents, whereas those on putative DETCs in other animals are not invariant, but display limited diversity.

## Author Contributions

YS and RM conducted experiments which formed part of the arguments made in this paper. YS and MK wrote the paper.

## Conflict of Interest Statement

The authors declare that this paper was written in the absence of any commercial or financial relationships that could be construed as a potential conflict of interest.
